# Lessons from elective in vitro fertilization (IVF) in, principally, non-infertile women

**DOI:** 10.1186/1477-7827-10-48

**Published:** 2012-06-20

**Authors:** Norbert Gleicher, Ann Kim, Andrea Weghofer, David H Barad

**Affiliations:** 1 Center for Human Reproduction, New York, NY, 10021, USA; 2 Foundation for Reproductive Medicine, New York, NY, 10021, USA; 3 Department of Gynecologic Endocrinology and Reproductive Medicine, Medical University Vienna, 1090, Vienna, Austria

**Keywords:** In vitro fertilization (IVF), Preimplantation genetic diagnosis (PGD), Preimplantation genetic screening (PGS), Aneuploidy, Pregnancy rates, Gonadotropin dosage, Anti-Müllerian hormone (AMH), Single embryo transfer

## Abstract

**Background:**

We here report the first investigation of exclusively elective in vitro fertilization (IVF) cycles in women with no apparent history of infertility. Since IVF outcome in women with infertility are always influenced by underlying causes of infertility, a study on non-infertile women may offer new insights.

**Methods:**

We investigated 88 females without history of infertility in 109 consecutive elective IVF cycles, almost exclusively performed for purposes of preimplantation genetic screening (PGS; i.e., elective gender selection). The following questions were addressed: (i) impact of PGS on IVF pregnancy chances; (ii) impact of transfer of 1 vs. ≥2 embryos on IVF pregnancy chances; (iii) correlation of anti-Müllerian hormone (AMH) levels to embryo ploidy (iv) effect of gonadotropin dosage used in stimulation on available embryos for transfer; and (v) in form of a 1:1 case control study, compared 33 elective PGS cycles with matched control cycles without PGS, performed in couples with either prior tubal ligations and/or severe male factor infertility as indication of IVF.

**Results:**

The overall clinical pregnancy rate for the group was 36.7%; pregnancy was associated with number of euploid (P = 0.009) and number of embryos transferred (P = 0.001). Odds of pregnancy were 3.4-times higher if ≥4 euploid embryos were produced in comparison to <4 (95% CI 1.2 to 9.2; P = 0.019), and odds of pregnancy were 6.6-times higher if greater than or equal to 2 rather than <1 euploid embryos were transferred (95% CI 2.0 to 21.7; P = 0.002). Increasing AMH (P = 0.001) and gonadotropin dosage used in ovarian stimulation (P = 0.024), was, independently, associated with number of available euploid embryos. Increasing AMH, but not follicle stimulating hormone (FSH), was associated with number of embryos available for biopsy and PGS (P = 0.0001). Implantation rates were 26.4% with PGS and 9.5% without (P = 0.008). Women undergoing PGS, demonstrated 4.58-times higher odds of pregnancy than matched controls (95% CI 1.102 to 19.060, Exp 4.584, P = 0.036).

**Conclusions:**

This study suggests that outcomes of elective IVF cycles may significantly deviate from infertility-associated cycles. Affirming proof of concept for PGS, utilizing day-3 embryo biopsy and fluorescence in-situ hybridization (FISH), both widely held responsible for earlier failures to establish such proof, suggests that the principal cause of prior failures were likely not insufficient laboratory techniques but poor patient selection for PGS. Such a conclusion questions the current reintroduction of PGS with improved techniques and technologies in absence of prior determination of suited patient populations.

## Background

Clinical utilization of in vitro fertilization (IVF) is almost exclusive to female and/or male infertility [1]. A rare exception is IVF in association with preimplantation genetic diagnosis (PGD), in, otherwise, presumed normally fertile women [2]. Such IVF cycles are usually assessed and reported as part of a center’s general IVF outcome statistics [3].

IVF cycle outcomes can, however, be expected to vary depending on whether conducted in infertile women or women with presumed normal fertility. IVF studies in only non-infertile patient populations, however, do not exist since they are rare and, therefore, difficult to accumulate at single IVF centers.

Likely for the first time, this study, therefore, reports on a homogenous cohort of IVF cycles, exclusively performed for non-infertility associated indications. Analyzing such cycles may allow for new insights into IVF without, otherwise, unavoidable patient biases from underlying causes of infertility.

One of the most important unresolved issues in IVF is whether selection of euploid embryo by eliminating aneuploid embryos before embryo transfer improves pregnancy rates and reduces miscarriage rates. To achieve this goal preimplantation genetic diagnosis, in this indication widely called preimplantation genetic screening, (PGS) [4], was widely utilized, until shown to be largely ineffective, and, indeed, reducing pregnancy chances in older infertile women [5-8]. Largely lacking underlying causes of infertility, a non-infertile patient population may, however, be better suited to assess the validity of PGS.

To assess the value of PGS for embryo selection in IVF appears of utmost importance because, despite current consensus that PGS is ineffective [5-8], PGS is still widely utilized for this indication. This study, therefore, attempted to utilize elective IVF cycles in non-infertile women to assess the value of PGS within a concept of embryo selection, and for a number of other unresolved issues in IVF relating to embryo ploidy.

## Methods

The study reports on 88 consecutive women undergoing 109 elective IVF cycles involving preimplantation genetic diagnosis (PGD) for fertility-unrelated indications (i.e., PGS).

### Patient selection

Our center voluntarily follows U.S. national guidelines for PGS, including most recent opinions of the Ethics Committee of the American Society of Reproductive Medicine (ASRM) in regards to elective gender selection, as summarized by Robertson in 2003 [9], and published in updated form by the Committee in 2004 [10]. This means that IVF is not performed solely for purposes of gender selection.

IVF is offered in association with PGD for medical indications, including single gene disorders, parental translocations, sex-linked diseases, and in medico-social circumstances, when mental health care providers determine that circumstances warrant gender selection. Our center, for example, serves a large gay community, where a desire for gender selection is common, and supported by the mental health community. The study, however, also includes one heterosexual, single female with psychiatric disease, where psychiatric clearance strongly recommended gender selection for female.

If other medical reasons exist for IVF, gender selection by PGS may be added if requested by the patient. Examples include tubal ligation or clinical indications for PGS (i.e., repeated unexplained aneuploidies in prior pregnancies). We currently do not offer PGS for purposes of embryo selection to improve IVF pregnancy chances and/or reduce miscarriage rates. In accordance with opinions expressed by the Ethics Committee of ASRM [10], we have been offering gender selection in non-medical cases only for family balancing purposes.

In this study we included only women undergoing IVF + PGS for the purpose of gender selection. Almost all cases involved elective gender selection for family balancing purposes but 12 cases involved psychiatric recommendations and three sex-linked diseases. Cycles where PGD was performed for single gene diseases were excluded.

PGD involves analysis of single blastomeres for chromosomal abnormalities and/or single gene diseases. As noted, when with the intent of reducing miscarriage risk and increasing pregnancy chances used to assess embryo ploidy, the procedure is now described under the acronym PGS [8].

To qualify for inclusion in this study, women had to undergo PGS without evidence of a concomitant infertility diagnosis. A history of tubal ligation or male factor infertility was, however, permitted since neither affects IVF outcomes. In cases of semen abnormalities, our center uniformly utilizes intracytoplasmic sperm injection (ICSI) to maximize embryo yields, thus eliminating potential male factor issues.

Fertility-reducing pathologies in study patients can, however, not be ruled out. Indeed, a degree of unknown infertility can be expected in a population, which has not attempted to conceive. Whatever infertility exists can be expected to be moderate in degree since PGS requires minimum embryo numbers, thus precluding significantly diminished ovarian reserve (DOR). Milder degrees of DOR, however, had to exist in a significant number of patients as abnormal anti-Müllerian hormone (AMH) and follicle stimulating hormone (FSH) levels demonstrate (Table 1).

**Table 1:** Comparison of patient characteristics and IVF cycle outcomes for both patient cohorts

	**Total cohort**	**Sub-cohort**
*Patients (n)*	88	53
*Age (years)*	36.8 ± 5.0	35.1 ± 4.5
*IVF cycles (n)*	109	69
* Embryos biopsied (n)*	7.0 ± 3.6	6.3 ± 3.5
* Euploid embryos (n)*	3.6 ± 2.3	3.4 ± 1.0
*Embryos transferred (n)*	1.7 ± 0.7	1.4 ± 1.0
*BMI*	n/a	23.8 ± 3.7
*AMH (ng/mL)*	n/a	2.3 ± 2.1
*FSH (mIU/mL)*	n/a	9.8 ± 3.8
*Estradiol (pg/mL)*	n/a	47.1 ± 26.0
*Total gonadotropin dosage (IU)*		4637 ± 2078

Both patient cohorts did not differ in age and IVF cycle outcomes.

### Sub-study group

For 53 patients, undergoing 69 IVF cycles, complete sets of AMH, FSH and estradiol data were available prior to first IVF cycle start, as well as total gonadotropin stimulation dosages. This sub-set of patients/cycles was used to determine how AMH and FSH, as markers of functional ovarian reserve, and gonadotropin dosage used for ovarian stimulation, relate to embryo ploidy.

### Control group to assess effectiveness of PGS

To assess potential effects of PGS on pregnancy chances, a case control group was required, which did not undergo embryo biopsy and PGS. Controls were selected from the center’s infertility patient pool but had to have undergone IVF with principal indication of male factor infertility. This control group was chosen to isolate, within the PGS procedure, the potential negative effects of embryo biopsy on implantation chances, while preserving potential beneficial effects from embryo selection. Women with secondary diagnoses of polycystic ovarian syndrome (PCOS) or tubal infertility, were, therefore, disqualified to preserve controls devoid of potentially additive female infertility causes that could contribute to lower implantation/pregnancy rates.

In addition, age of controls had to be within ± 1 year of study patients, and ovarian reserve had to be similar, defined as oocyte yields within ± 2 oocytes. To control for the laboratory environment and changes in clinical protocols, all cycles had to be conducted after 2008 since no significant staff and/or protocol changes have occurred since.

These strict matching criteria only yielded 25 women, undergoing 33 IVF cycles (from 109 elective cycles) and a control group of 28 women. Both groups underwent 33 IVF cycles (25 study patients 1 cycle each, and 4 having 2 cycles), while amongst 28 controls, 28 had 1 cycle, 1 had 2, and another 1 underwent 3 cycles.

### PGS laboratory technique

Since here presented data represent a retrospective analysis, the methodology utilized to perform PGS involved standard techniques and technologies utilized worldwide during the study period. This means that PGS was performed after day-3 embryo biopsy (6–8 cell stage). A single blastomere was removed and examined by fluorescence in situ hybridization (FISH) for seven chromosomes (X, Y, 13, 16, 18, 21, 22).

It, therefore, is important to point out that here utilized techniques and technologies are the same as widely utilized during initial failed attempts at introduction of PGS for embryo selection, with purpose of improving IVF pregnancy rates [5-8].

### Clinical cycle management

Ovarian stimulation was uniform: Patients with normal age-specific ovarian reserve were down-regulated with luteal phase agonist (leuprolide acetate, Lupron™, Abbot Pharmaceutical, North Chicago, IL) and stimulated with between 225–450 IU of follicle stimulating hormone and human menopausal gonadotropin (hMG), both from different manufacturers. Patients with abnormal ovarian reserve received microdose agonist (Lupron™), followed by the same combination of gonadotropins but with maximal combined dosage of 600 IU daily. All patients received gonadotropin as FSH, except for 150 IU, given as hMG.

Normal versus abnormal ovarian reserve was determined for each patient/cycle based on previously reported normal age-specific ranges for FSH [11] and AMH [12].

Only clinical intrauterine pregnancies were considered, with exclusion of chemical pregnancies and ectopic pregnancies from statistical consideration. A clinical pregnancy was defined as a normally developing intrauterine pregnancy with fetal heart, seen on ultrasound.

### Statistical analysis

Since 109 cycles in 88 women were distributed amongst 69 patients with one, 17 with two, and two with three IVF cycles, all statistical models were adjusted for repeat cycles.

Categorical data were expressed as numbers and percentages, and numerical data as mean ± standard deviation (SD), except when specified. Student’s *t*-test, chi - square and Fisher’s exact test were used as appropriate. Associations of cycle characteristics with outcomes of interest were evaluated using logistic regression analyses, and are reported as odds ratios (OR) and 95% confidence intervals (95% CI). Significance was defined as P < 0.05.

Multivariate logistic regression analyses were performed to determine independent predictors for clinical pregnancy. Covariates were included in the adjusted models, based on evidence of statistical significance to an association with a respective outcome on univariate analysis or when plausibility for such a relationship was obvious. For example, since in the case–control part of this study gonadotropin dosages and embryo numbers varied to a significant degree between both groups, both were included as covariates in the model.

All statistical analyses were performed using the Statistical Package for Social Science, version 19.0 (SPSS Inc., Chicago, IL)

### Institutional review board

The center’s Institutional Review Board (IRB), for this study required only expedited review since it involved only retroactive data/chart review. All patients presenting to our center sign at time of first visit an informed consent, which allows for use of their medical record for research purposes, as long as the patients’ anonymity remains protected and the medical record remains confidential. Both conditions were met here. In addition, the center’s research staff with access to medical records is under federal HIPAA rules in writing committed to confidentiality. Written informed consents are available for review by the Editor-in-Chief of this journal.

## Results

Table 1 summarizes patient data for all 88 patients and 109 IVF cycle: mean age was 36.8 ± 5.0 years; an average of 7.0 ± 3.6 embryos were biopsied, of which approximately half (3.6 ± 2.3) proved euploid; 1.7 ± 0.7 embryos were transferred.

Table 2 demonstrates that 109 IVF cycles led to 40 pregnancies (36.7%). Pregnant women were non-significantly younger than of those who failed to conceive. Number of embryos biopsied also did not differ between pregnant and non-pregnant patients.

**Table 2:** IVF cycle characteristics for pregnant versus non-pregnant patients

	**Pregnant**	**Not pregnant**
*Percent of total cycles*	36.7	63.3
*Age (years)*	36.5 ± 5.3	37.0 ± 4.6
*Embryos biopsied (n)*	7.7 ± 3.7	7.0 ± 3.8
* ≤ 6 n (%)*	20 (50.0)	35 (50.7)
* ≥ 7 n (%)*	20 (50.0)	34 (49.3)
*Euploid embryos (n)*^*1*^	4.5 ± 2.4	3.3 ± 2.2
* ≤ 3 n (%)*	16 (40.0)	42 (60.9)
* % pregnant*	27.6	
* ≥ 4 n (%)*	24 (60.0)	27 (39.1)
* % pregnant*	47.1	
*Embryos transferred (n)*	2.1 ± 0.7	1.6 ± 0.8
*≥ 2 euploid embryos available*^*2*^		
* n (%) pregnant ET x 1*	4/35 (11.4)	
* n (%) pregnant ET x 2*	36/74 (48.6)	

Pregnant and non-pregnant women neither differed in age nor in number of embryo biopsied. ^1^Logistic regression for pregnancy, adjusted for age and depending on whether ≥4 or ≤3 euploid embryo were present in cycle, adjusted by number of embryos biopsied and transferred, demonstrated over 3 fold pregnancy odds in women who had ≥ 4 euploid embryos (95% CI 1.224 to 9.201, Exp 3.356, P = 0.019). ^2^Logistic regression analysis, adjusted for age cycle number, euploid embryos and depending on whether ≤1 or ≥2 euploid embryo were transferred demonstrated 6.553-times the odds of pregnancy with transfer of ≥2 embryos (95 CI 1.979 to 21.695, Exp 6,553, P = 0.002).

### Pregnancy chances based on ploidy

**Figure 1:**
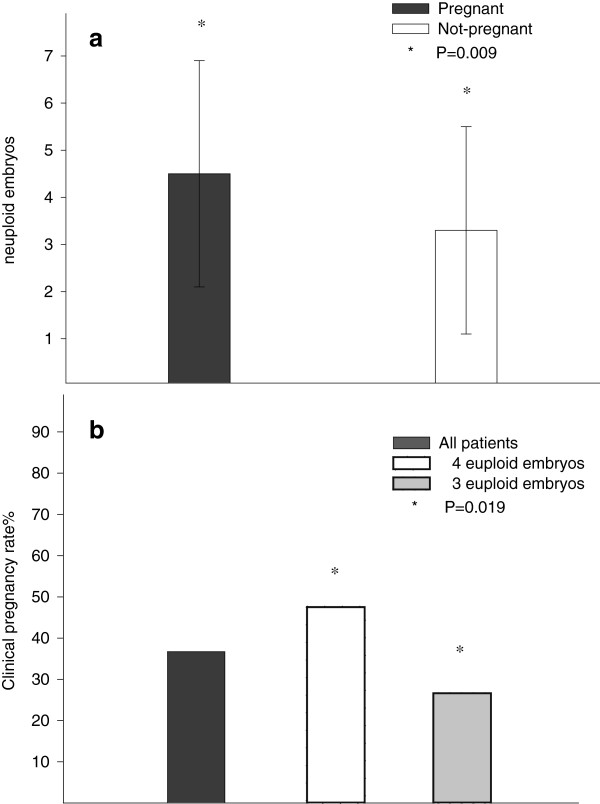
**Clinical pregnancy rates depending on number of euploid embryos generated in IVF cycle.** Figure 1a demonstrates that patients who achieved pregnancy produced significantly more euploid embryos (4.5 ± 2.4 vs. 3.3 ± 2.2, P = 0.009), while 1**b** demonstrates that patients who in their IVF cycles produced ≥4 euploid embryos, with transfer of similar embryo numbers, produced a 47.5% clinical pregnancy rates, while patients who only produced ≤3 euploid embryo in their cycle achieved only a 26.6% clinical pregnancy rate, creating for women with ≥4 euploid embryos 3.4-times higher odds of pregnancy (95% CI 1.2 to 9.2; P = 0.019).

As shown in Table 2, pregnant patients, however, produced significantly more euploid embryos (4.5 ± 2.4 vs. 3.3 ± 2.2; P = 0.009). Logistic regression for clinical pregnancy, adjusted for age, number of embryos biopsied and transferred found that patients who had produced ≥4 euploid embryos demonstrated 3.4-times the odds of pregnancy in comparison to those with ≤3 euploid embryos (95% CI 1.2 to 9.2; P = 0.019). This translated into a clinical pregnancy rate of 47.1% if a cycle resulted in ≥4 euploid embryos (even if fewer embryos were transferred, as will be demonstrated below) versus 27.6% with ≤3 embryos (Figure 1)

### Pregnancy chances based on number of embryos transferred

Pregnant patients received significantly more embryos (2.1 ± 0.7 vs. 1.6 ± 0.8; P = 0.001). Logistic regression, adjusted for age, attempted cycle number, number of euploid embryos and depending on whether ≤1 or ≥2 embryos were transferred, demonstrated 6.6-times the odds of pregnancy with transfer of ≥2 embryos (95% CI 2.0 to 21.7; P = 0.002). For statistical reasons patients had to be classified as receiving ≤1 or ≥2 embryos, though no patient, of course, received less than one embryo.

### Factors associated with embryo ploidy

Table 1 also demonstrates that there was no difference in cycle characteristics between the whole study cohort of 88 women (and their 109 cycles) and the smaller study cohort for which AMH, FSH and dosage of gonadotropin stimulation, were available (53 women, 69 cycles).

This smaller patient group allowed construction of a model to determine associated patient factors with embryo euploidy. In a model, adjusted for number of embryos biopsied, AMH and patient age, increasing AMH levels and increasing gonadotropin dosages were significantly associated with increasing numbers of euploid embryos (AMH, P = 0.001; gonadotropin dosage, P = 0.024). AMH (P = 0.0001) but neither FSH nor estradiol related to number of embryos biopsied.

### Effectiveness of PGS

As Table 3 demonstrates, in the case–control study both groups were well matched: Ages were 38.7 ± 4.0 and 39.2 ± 4.0 years (range 27.1–45.0 and 27.7–45.4); oocyte yields 9.7 ± 5.6 and 9.7 ± 5.4 (range for both groups, 2–25); AMH values were 1.5 ± 1.4 and 1.4 ± 1.8 ng/mL, respectively. None of these values differed between the two groups.

**Table 3:** Patient/cycle characteristics for case–control study

	**PGS Group**	**Control Group**
*n IVF cycles*	30	30
*Age (years)*	38.7 ± 4.0	39.2 ± 4.0
*Range*	27.1–45.0	27.7–45.4
*AMH (ng/mL)*	1.5 ± 1.4	1.4 ± 1.8
*Total gonadotropin dosage (IU)*^*2*^	4840 ± 1825	5934 ± 2330
*Oocyte yields (n)*	9.7 ± 5.6	9.7 ± 5.4
*Range*	2–25	2–25
*Embryos transferred (n)*^*1*^	1.6 ± 0.8	2.9 ± 1.0
*Implantation rate (%)*^*3*^	26.4	9.5
*Heartbeats/Embryos transferred*	14/53	9/95
*Pregnancy rate (%)*^*4*^	36.4	21.2
*n*	12/33	7/33
*Miscarriage rate (%)*	8.3	14.3
*n*	1/12	1/7

^1^ Controls had significantly more embryo transferred (P < 0.0001), and ^2^ utilized marginally more total gonadotropins (P = 0.046). ^3^ The implantation rate was significantly higher in PGS cycles (P = 0.008). ^4^ In univariate analyses pregnancy rates and miscarriage rates did not differ significantly. After appropriate adjustments, logistic regression analysis demonstrated 4.58-times the odds of clinical pregnancy after PGS than in controls (95% CI 1.102 to 19.060, Exp 4.584; P = 0.036).

Total gonadotropin dosages for ovarian stimulation were 4840 ± 1825 IU in PGS patients and 5934 ± 2330 IU in controls, a marginally larger dose in the latter group (P = 0.046). Controls also received significantly more embryos (2.9 ± 1.0 vs. 1.6 ± 0.8; P < 0.0001).

Table 3 also summarizes implantation, pregnancy and miscarriage rates: Amongst PGS patients, 14/53 (26.4%) of transferred embryos implanted; in control cycles only 9/95 (9,5%; P = 0.008). Pregnancy rates were higher in PGS (12/33, 36.4%) than control patients (7/33, 21.2%). Likely due to small cycle numbers, unadjusted, this difference did not reach significance. Similarly, miscarriage rates were lower in PGS cycles (1/12, 8.3%) vs. controls (1/7, 14.3%) but, likely, because of low miscarriage rates, this difference did not reach statistical significance, either.

In PGS cycles logistic regression analysis, adjusted for age, number of prior IVF cycles, number of embryos transferred and gonadotropin dosage, demonstrated, however, 4.58-times the odds of achieving a clinical pregnancy in comparison to controls (95% CI 1.102 to 19.060, P = 0.036).

## Discussion

Patient ages, FSH, AMH and gonadotropin dosages, utilized for ovarian stimulation, demonstrate that here investigated patients are neither especially young nor do they have unusually favorable ovarian reserve. They, indeed, appear to represent a rather unfavorable middle-aged patient population. Especially if not having attempted conception, as usually the case in couples desirous of elective gender selection, they will not be devoid of fertility problems. They, however, still, can be expected to differ from standard infertile populations, and, therefore, should demonstrate distinct IVF outcome variations. Their investigation may, therefore, offer interesting new information.

Though in humans never been proven, improved embryo aneuploidy should better IVF pregnancy rates, [8,13]. We previously suggested that a principal reasons why PGS so far has failed to demonstrate improvements in IVF outcomes has been poor patient selection [13]. Because aneuploidy increases with advancing female age, older women, have been widely considered the best candidates for PGS [14]. In older women, PGS, however, was actually demonstrated to reduce pregnancy chances [7,15]. How can that be?

As noted in the introduction, the principal purpose of PGS is embryo selection. Older women, however, usually no longer produce large enough embryo numbers to warrant embryo selection [13]. They, therefore, end up with all the downsides of PGS without any of its benefits: decreased implantation/pregnancy chances from embryo biopsies but no compensatory benefits from embryo selection.

Considering here studied patients’ ages, their ovarian reserves (Table 1), that all embryos underwent biopsy for PGS, known to reduce pregnancy chances [13,16], and that gender selection results in “loss” of approximately half of all embryos (the undesired sex), the observed clinical pregnancy rate of 36.7% per cycle start is not only excellent but reflective of the national average IVF pregnancy rate reported in Annual Centers for Disease Control (CDC) reports. Here utilized patients, therefore, appear well suited to investigate effects of ploidy in IVF.

This is further supported by here reported cycle characteristics, for example that women who conceived were only insignificantly younger than those who did not, had similar numbers of embryos biopsied and, therefore, demonstrated no significant differences in oocyte and embryo yields.

Yet, women who did conceive produced significantly more euploid embryos (4.5 ± 2.4 vs. 3.3 ± 2.2, P = 0.009). Availability of ≥4 embryos, in comparison to <4, resulted in 3.4-times the chance of pregnancy (P = 0.019; Figure 1), though much fewer embryos were really transferred (2.1 ± 0.7 in cycle leading to pregnancy and 1.6 ± 0.8 in failed cycles).

All factors being equal, some women, therefore, have better IVF pregnancy chances because they produce more euploid embryos than others, independent of age. Those embryos also exhibit higher pregnancy potential. The conclusion is that embryo quantity, in principle, runs in parallel with embryo quality, though specific conditions, like polycystic ovaries or age, may favor one or the other. Effectiveness of PGS should, therefore, vary in different patient populations [13].

In cycles leading to pregnancy significantly more embryos were transferred (2.1 ± 0.7 vs. 1.6 ± 0.8; P = 0.001). Logistic regression, adjusted for relevant confounders, confirmed the importance of numbers of transferred embryos: Cycles with ≥2 resulted in 6.6-times the pregnancy chance of cycles with only one transferred embryo (P = 0.002), an observation with considerable relevance to the currently ongoing debate about single embryo transfer [17]. Here presented data confirm that single embryo transfer significantly reduces pregnancy chances in comparison to two-embryo transfers [18].

Increasing AMH levels (P = 0.001) and gonadotropin dosages (P = 0.024) were, independently, associated with increasing proportions of euploid embryos (i.e., increasing number of normal embryos available for potential transfer). The AMH association supports reports that AMH reflects functional ovarian reserve quantitatively [12] as well as qualitatively [19].

This suggests that FSH and/or AMH ratios per oocyte or embryo could be predictive of ploidy in eggs and embryos. We recently, indeed, demonstrated that a ratio of FSH per oocytes (FSHo) at all ages is constant and highly predictive of pregnancy with IVF [20]. The potential benefit of such ratios is also suggested by declining AMH production per oocyte with advancing female age [21], and variations of AMH levels per oocyte between races/ethnicities [22]. Finally, the association of rising AMH with improving ploidy is also supported by dehydroepiandrosterone (DHEA) supplementation in women with DOR [23], leading to lower embryo aneuploidy [24] miscarriage rates [25] and higher pregnancy and live birth chances [26-28].

Here observed positive association between increasing total gonadotropin dosage for ovarian stimulation and improving proportions of available euploid embryos for embryo transfer is important because it contradicts published literature in two ways: Some authors, in contrast to our data, have associated increasing gonadotropin dosages with increasing aneuploidy rates [29,30]; and others have claimed that oocyte and embryo yields no longer improve beyond a rather low ceiling in gonadotropin stimulation dosage [31]. These differences may, indeed, be reflective of differences in investigated patient populations. Adverse effects of gonadotropin stimulation on ploidy should not be considered established: In patients, who spontaneously or after FSH stimulation conceived, ploidy did not differ [32].

Since daily ovarian stimulation in this study included 150 IU of hMG, here reported findings support previously reported data (in long agonist protocols) demonstrating that luteinizing hormone (LH)-containing stimulation improves ploidy versus stimulation with pure FSH [33]. FSH, and indirectly LH (through control over the follicle’s androgen production in theca cells) [34,35], plays an essential role in early stages of follicle development. Longer and/or higher gonadotropin exposure may, therefore, beneficially affect follicle maturation. Just as androgens used to be viewed as damaging to follicle maturation, and are now recognized as essential [36,37], gonadotropins may, therefore, actually be beneficial to developing follicles and oocytes.

Improving ploidy in transferred embryos has been the main argument in favor of PGS [4]. Based on PGD/PGS pioneers, like Kuliev and Verlinsky, recommending that PGS become integral to routine IVF [38], centers around the world, indeed, initiated routine PGS use until a Dutch randomized study proved PGS ineffective, and in older women actually demonstrating that it reduces pregnancy chances [39]. Since then, utilization of PGS in attempts of improving IVF pregnancy chances has been widely discouraged [5-8].

The failure of PGS was widely attributed to inadequate procedural techniques, especially embryo biopsy (day-3 removal of 1–2 blastomers) and aneuploidy detection by fluorescent in-situ hybridization (FISH) [16]. Others, however, pointed towards poor patient selection as principal cause [8,13]. Currently ongoing active efforts at reintroducing PGS into routine IVF, this time utilizing improved techniques and technologies [40], stresses the urgency of resolving this conflict, as any such reintroduction is, likely, bound to fail again if patient selection, indeed, proves to have been the decisive factor in the earlier PGS failure.

The case–control part of this study was designed to resolve this issue: If, indeed, inadequate techniques and technologies had been responsible for the initial PGS failure, we should be unable to establish proof of concept for PGS, utilizing FISH and day-3 embryo biopsy in this study. Confirmation proof of concept for PGD would, however, strongly suggest that patient selection was the cause.

In a retroactive analysis, elective IVF cycles require well-matched controls [41]. Choosing rigidity of the matching process over study size, only 33 of originally 108 elective IVF cycles could be properly matched. Here reported statistically significant results, however, validate this approach. Though small in size, this sub-study, nevertheless, confirmed proof of concept: Embryo selection by PGS, indeed, improved embryo implantation rates and pregnancy chances by demonstrating that in age-matched patient cycles (with identical ovarian reserve), implantation rates were significantly higher after PGS (P = 0.008).

Likely due to the small study size, pregnancy rates in univariate analysis missed significance. Appropriate statistical adjustments for number of embryos transferred and gonadotropin dosages (both significantly different by univariate analysis, Table 3) and repeat cycles, demonstrated, however, that the odds of pregnancy were actually 4.58-times higher after PGS (P = 0.036).

Considering the small study size, these results are surprisingly robust statistically. Since definition of miscarriage required prior fetal heart activity, and so-called missed abortion, therefore, were not considered, it would be unrealistic to expect significant results in regards to pregnancy loss. Missed abortions in a majority, represent aneuploid pregnancies [42]. Their inclusion, therefore, even in this small study may also have demonstrated a statistical benefit from PGS on pregnancy loss. This is supported by the observed 14.3% miscarriage rate in controls, representing the expected miscarriage rate in a general population after spontaneous conception [43], while PGS patients, at 8.3%, demonstrated almost half the rate.

This first study ever exclusively performed in elective IVF cycles, thus, offers new information on a number of important unresolved issues: (i) Embryo ploidy varies between women, even when controlled for covariates, such as age and ovarian reserve. Moreover, since pregnancy potential of euploid embryos also varies between women, ploidy is not the only pregnancy-determining factor; (ii) Single embryo transfer significantly reduces pregnancy chances in comparison to ≥2 embryos; (iii) Increasing AMH levels reflect increasing euploid embryo numbers, confirming AMH as a quantitative as well as qualitative assessment; (iv) Available euploid embryos actually increase with increasing gonadotropin dosages, suggesting that up to 600 IU daily may contribute to better IVF pregnancy rates; and (v) Embryo selection via PGS in properly selected patients can, indeed, improve embryo implantation and IVF pregnancy rates and will, likely, also reduce miscarriage rates.

Confirmation of proof of concept for PGS with use of traditional techniques and technologies suggests that the original worldwide clinical failure of PGS was based on patient selection, and not inadequate techniques and technologies. Newly proposed embryo biopsy timing (trophectoderm biopsy day-5, blastocyst-stage) and improved ploidy assessment technologies (arrays, offering assessments of all chromosomes), therefore, will be unlikely to beneficially affect the clinical utility of PGS in association with IVF.

One, of course, would have preferred a prospectively randomized study having led to these conclusion but prospectively randomized studies in IVF are often very difficult, if not impossible, to perform [41]. An eagerly anticipated clinical trial of PGS under the auspices of the European Society for Human Reproduction and Embryology (ESHRE) has been announced [44]. Until results of properly conducted clinical trials become available, other study formats have to be accepted as best available evidence [41]. Here presented data in form of a rigorous case control study, currently, therefore represent best available evidence, even in comparison to larger, uncontrolled studies.

Any retroactive analysis of outcome data is, of course, open to unrecognized biases. While non-infertile women in elective IVF cycles avoid many selection biases of infertile patients, even such a patient population cannot avoid all biases.

Further studies are required to better define patient populations, which will benefit from PGS. To prevent a second premature introduction of PGS, the procedure experimental, and should be offered only with experimental informed consent, and under study conditions.

## Conclusions

This study for the first time presents an IVF experience of exclusively elective not-infertility related cycles. Such cycles in some aspects vary from what has been reported for IVF cycles in infertile women, suggesting that, in interpreting IVF outcomes, current literature may not always properly differentiate between outcome contributions from underlying infertility and the IVF procedure, itself. Utilizing day-3 embryo biopsy and FISH, the study also established proof of concept that, in properly selected patients, PGS, indeed, improves pregnancy chances with IVF and, likely, reduces miscarriage risk. This observation suggests that earlier failures in proving the efficacy of PGS were, likely not the result of poor techniques and technologies, as has been suggested, but the consequence of poor patient selection.

## Abbreviations

AMH: Anti-Müllerian hormone; DHEA: Dehydroepiandrosterone; DOR: Diminished ovarian reserve; FISH: Fluorescence in situ hybridization; FSH: Follicle stimulating hormone; hMG: Human menopausal gonadotropin; ICSI: Intracytoplasmic sperm injection; IVF: In vitro fertilization; LH: Luteinizing hormone; PCOS: Polycystic ovarian syndrome; PGD: Preimplantation genetic diagnosis; PGS: Preimplantation genetic screening.

## Competing interests

N.G, A.W. and D.H.B. have in the past received research support, speakers’ honoraria and travel funds from various pharmaceutical and medical device companies, none, however, related to the subject of this paper. N.G and D.H.B, are listed as co-inventors of two awarded U.S. patents, claiming therapeutic benefits for DHEA, and potentially other androgens, in women with DOR. Both authors have other pending patent applications, regarding DHEA, and other androgens, and, unrelated to this presentation, the *FMR1* gene’s effects on ovaries. N.G. owns shares in Fertility Nutraceuticals, LLC, a company that sells a DHEA product. N.G. and D.H.B. are receiving patent royalties from this company. N.G. is also the owner of The CHR, where this research was conducted.

## Authors’ contributions

N.G. and D.H.B., overall, contributed equally to the manuscript. N.G. wrote the manuscript, D.H.B. performed data analysis. A.K. contributed to data and statistical analyses. A.W. contributed to study design. All authors read and approved the final manuscript.
